# Continuing professional development system for health-care professions, Egypt

**DOI:** 10.2471/BLT.22.287963

**Published:** 2022-04-26

**Authors:** Mohamed Reda Bassiouny, Azza R Elhadidy

**Affiliations:** a School of Medicine, Mansoura University, PO Box 35, Mansoura University, Mansoura, 35516, Egypt.

## Abstract

While the regulatory framework for medical education in Egypt has rapidly evolved, the progress of developing a system for continuing professional development has been slow. In 2018 the government approved legislation establishing a regulatory authority for continuing professional development and added expectations for continuing professional development as a condition of relicensure for physicians in Egypt. The new authority has deployed a provider-accreditation model that sets criteria for educational quality, learning outcomes, independence from industry, and tracking of learners. Only accredited providers can submit continuing professional development accredited activities. Despite regulatory and administrative support there have been several barriers to the implementation of the system including limited availability of funding, lack of suitable training venues and equipment for hands-on training, and resistance from the profession. As of March 2022, 112 continuing professional development providers have achieved accreditation, and deployed 154 accredited continuing professional development activities. The majority of accredited providers were medical associations (64%) and higher education institutions (18%), followed by medical foundations and nongovernmental organizations (13%) and health-care facilities (5%). One electronic learning platform has been accredited. Any entity with commercial interests cannot be accredited as a continuing professional development provider. Funding of continuing professional development activities can be derived from provider budgets, programme registration fees or appropriate sponsors. Funding from industry is limited to unrestricted educational grants. The foundations for an effective continuing professional development system have been established in Egypt with the aim of achieving international recognition.

## Résumé

Si le cadre réglementaire de l’enseignement médical en Égypte a rapidement évolué, la progression de l’élaboration d’un système de développement professionnel continu a été lente. En 2018, le gouvernement a approuvé la législation établissant une autorité de réglementation en faveur du développement professionnel continu et a ajouté des attentes en matière de développement professionnel continu comme condition de renouvellement du permis d’exercer pour les médecins en Égypte. Cette nouvelle autorité a diffusé un modèle d’accréditation des prestataires qui fixe des critères de qualité éducative, de résultats d’apprentissage, d’indépendance vis-à-vis du secteur et de suivi des apprenants. Seuls les prestataires accrédités peuvent présenter des activités agréées de développement professionnel continu. Malgré un soutien réglementaire et administratif, plusieurs obstacles ont entravé la mise en œuvre de ce système, notamment la disponibilité limitée de fonds, le manque de lieux de formation et d’équipements adaptés à une formation pratique, ainsi que la résistance de la part de la profession. En mars 2022, 112 prestataires de formation professionnelle continue avaient obtenu leur accréditation et mis en place 154 activités de formation professionnelle continue agréées. La majorité des prestataires accrédités étaient des associations médicales (64%) et des établissements d’enseignement supérieur (18%), suivis par des fondations médicales et des organisations non gouvernementales (13%) et des établissements de soins de santé (5%). Une plateforme d’apprentissage électronique a été accréditée. Toute entité ayant des intérêts commerciaux ne peut être accréditée en tant que prestataire de services de développement professionnel continu. Le financement des activités de développement professionnel continu peut provenir des budgets des prestataires, des frais d’inscription aux programmes ou de sponsors appropriés. Le financement par le secteur se limite à des subventions éducatives sans restriction. Les bases d’un système efficace de développement professionnel continu ont été établies en Égypte dans le but d’obtenir une reconnaissance internationale.

## Resumen

Mientras que el marco regulatorio para la educación médica en Egipto ha evolucionado con rapidez, el progreso del desarrollo de un sistema para el desarrollo profesional continuo ha sido lento. En 2018, el gobierno aprobó la legislación que establece una autoridad reguladora para el desarrollo profesional continuo y añadió las expectativas de desarrollo profesional continuo como condición para la renovación de la licencia de los médicos en Egipto. La nueva autoridad ha desplegado un modelo de acreditación de proveedores que establece criterios de calidad educativa, resultados de aprendizaje, independencia del sector y seguimiento de los estudiantes. Solo los proveedores autorizados pueden presentar actividades acreditadas de desarrollo profesional continuo. A pesar del apoyo normativo y administrativo, ha habido varios obstáculos para la aplicación del sistema, como la escasa disponibilidad de fondos, la falta de centros de formación y equipos adecuados para la formación práctica, y la resistencia de la profesión. En marzo de 2022, 112 proveedores de desarrollo profesional continuo han logrado la acreditación y han desplegado 154 actividades de desarrollo profesional continuo acreditadas. La mayoría de los proveedores autorizados eran asociaciones médicas (64 %) e instituciones de enseñanza superior (18 %), seguidas de fundaciones médicas y organizaciones no gubernamentales (13 %) y centros de atención sanitaria (5 %). También se ha acreditado una plataforma de aprendizaje electrónico. Las entidades con intereses comerciales no pueden ser acreditadas como proveedores de formación profesional continua. La financiación de las actividades de desarrollo profesional continuo se puede obtener de los presupuestos de los proveedores, de las cuotas de inscripción en los programas o de los patrocinadores correspondientes. La financiación obtenida del sector se limita a subvenciones educativas sin restricciones. En Egipto, se han establecido las bases de un sistema eficaz de desarrollo profesional continuo con el objetivo de lograr el reconocimiento internacional.

## ملخص

بينما يتطور إطار العمل التنظيمي للتعليم الطبي في مصر بسرعة، إلا أن التقدم في تطوير نظام للتطوير المهني المستمر كان بطيئًا. في عام 2018، وافقت الحكومة على تشريع لتأسيس هيئة تنظيمية للتطوير المهني المستمر، وإضافة توقعات للتطوير المهني المستمر، كشرط لإعادة منح الترخيص للأطباء في مصر. قامت الهيئة الجديدة بنشر نموذج اعتماد لمقدم الخدمة، والذي يحدد معايير جودة التعليم، ونتائج التعلم، والاستقلال عن المجال، وتتبع المتعلمين. يمكن فقط لمقدمي الخدمات المعتمدين تقديم الأنشطة المعتمدة للتطوير المهني المستمر. وعلى الرغم من الدعم التنظيمي والإداري، كانت هناك عدة حواجز أمام تنفيذ النظام بما في ذلك التوفر المحدود للتمويل، ونقص أماكن التدريب المناسبة ومعدات التدريب العملي، ومقاومة المهنة. اعتبارًا من مارس/آذار 2022، حصل 112 من مقدمي خدمة التطوير المهني المستمر على الاعتماد، ونشروا 154 نشاطًا معتمدًا للتطوير المهني المستمر. كانت غالبية مقدمي الخدمة المعتمدين من الجمعيات الطبية (64%)، ومؤسسات التعليم العالي (18%)، وتليها المؤسسات الطبية والمنظمات غير الحكومية (13%) ومنشآت الرعاية الصحية (5%). تم اعتماد منصة تعلّم إلكترونية واحدة. أي كيان له اهتمامات تجارية، لا يمكن اعتماده كمقدم لخدمة التطوير المهني المستمر. يمكن الحصول على تمويل أنشطة التطوير المهني المستمر من ميزانيات مقدمي الخدمة، أو رسوم تسجيل البرنامج، أو جهات الرعاية المناسبة. يقتصر التمويل من المجال على المنح التعليمية غير المقيّدة. تم وضع الأسس لنظام تطوير مهني مستمر فعال في مصر بهدف الحصول على الاعتراف الدولي.

## 摘要

虽然埃及医学教育的法规框架迅速发展，但建立继续医学教育体系的进展却很缓慢。印度政府于 2018 年批准了建立继续医学教育监管机构的立法，并期望将继续医学教育作为埃及医生取得再认证的条件。新成立的监管机构部署了一个教育机构认证模式，为教育质量、学习成果、部门的独立性以及学员跟踪设定标准。只有获得授权的教育机构才能提交继续医学教育认证活动。尽管有法规和行政方面的支持，但实施该系统仍然面临一些障碍，包括有限的可用资金、缺乏适合开展实操培训的培训场所和设备，以及同行抵制。截至 2022 年 3 月，已有 112 家继续医学教育机构获得授权，并开展了 154 项已认证的继续医学教育活动。已授权机构大多是医学会 (64%) 和高等教育机构 (18%)，其次是医学基金会和非政府组织 (13%) 以及医疗护理机构 (5%)。一个电子学习平台获得授权。任何具有商业利益的实体均不能被授权为继续医学教育机构。继续医学教育活动的资金可以来自机构的预算、项目注册费或适当的赞助费。来自部门的资金仅限于不受限制的教育补助金。埃及已为建立有效的继续医学教育体系打好基础，以望获得国际认可。

## Резюме

В то время как нормативно-правовая база медицинского образования в Египте развивалась быстро, прогресс в разработке системы непрерывного профессионального роста был медленным. В 2018 году правительство утвердило закон о создании регулирующего органа для осуществления непрерывного профессионального роста и тем самым добавило ожидания в отношении непрерывного профессионального роста как условия подтверждения разрешения на врачебную практику в Египте. Новый орган внедрил модель аккредитации поставщиков, которая устанавливает критерии качества образования, результатов обучения, независимости от отрасли и отслеживания обучающихся. Только аккредитованные поставщики могут представить аккредитованную деятельность по непрерывному профессиональному росту. Несмотря на нормативную и административную поддержку, на пути внедрения системы возникло несколько препятствий, включая ограниченное финансирование, отсутствие подходящих мест проведения обучения и оборудования для практического обучения, а также сопротивление со стороны работников. По состоянию на март 2022 года 112 поставщиков услуг в сфере непрерывного профессионального роста прошли аккредитацию и развернули 154 аккредитованных мероприятия по непрерывному профессиональному росту. Большинство аккредитованных поставщиков были медицинские ассоциации (64%) и высшие учебные заведения (18%), медицинские фонды и неправительственные организации (13%), а также учреждения здравоохранения (5%). Аккредитована одна электронная учебная платформа. Любая организация с коммерческими интересами не может быть аккредитована в качестве поставщика услуг в сфере непрерывного профессионального роста. Финансирование непрерывного профессионального роста может быть получено из бюджета поставщика, взносов за регистрацию программы или от соответствующих спонсоров. Финансирование со стороны отрасли ограничено неограниченными образовательными грантами. С целью достижения международного признания в Египте заложены основы эффективной системы непрерывного профессионального роста.

## Introduction

With over 102 million citizens in 2020 and high population growth, Egypt needs and deserves high-quality health care. Figures from 2018 show that Egypt had 40 medical schools, more than 2000 hospitals and 445 000 physicians working in the country,^1^ equating to around 7.4 doctors per 10 000 citizens. Egypt hosts hundreds of medical conferences annually, attracting tens of thousands of international attendees, especially from the Middle East and Africa. Many Egyptian doctors are working successfully worldwide. Nevertheless, progress has been slow in developing a continuing professional development system to support excellence in practice and care.^2^

Formal credit systems for continuing medical education were first established in 1948 in North America followed by Europe.^3^^,^^4^ From 1990 to 2011, several initiatives had sought to establish centres or systems for the accreditation of continuing professional development activities in Egypt.^2^ These initiatives included those of the Egyptian medical syndicate, the Egypt country office of the World Health Organization,^5^ medical scientific associations, the health ministry and several of the country’s medical schools. However, these initiatives have functioned without a national regulatory system, which has been the main challenge of continuing medical education in the country.^6^ None of these efforts were successful since physicians in Egypt had permanent or lifetime licensure so there was no incentive for them to engage in such development. Furthermore, the developing agencies and accreditors had conflicts of interest, while the lack of a national regulatory system meant that agencies sought to accredit themselves and their activities.^7^

In September 2017, the Higher Education Reform Experts in Egypt organized a workshop to discuss the planning, implementation and accreditation of continuing professional development in the health sector. The participants in the workshop included representatives from Egypt’s National Authority for Quality Assurance and Accreditation of Education (the standard-setting body for higher education), the Compulsory Egyptian Medical Training Authority (the authority in charge of continuing professional development accreditation), the relevant sector committees of the Supreme Council of Universities, higher education institutions, the health scientific societies, and the medical and nursing syndicates, as well as selected Egyptian politicians and European experts in the field.^8^ The workshop produced a recommendation that the Egyptian government designates a national licensing authority, requires relicensure and establishes an independent agency to accredit continuing professional development providers. We describe here the design and implementation of Egypt’s continuing professional development system for health-care professionals, which has begun with a focus on physicians before roll-out to other health professionals. 

## Legislative development

Before the workshop, the regulatory authority in charge of continuing professional development accreditation, the Compulsory Egyptian Medical Training Authority, was established by the Prime Minister’s decree no. 210/2016. Its governing board comprises members from higher education institutions, the health ministry, military and police medical services, and the Egyptian medical syndicate. The objectives of the regulatory authority included oversight of graduate and continuing medical education,^9^ including the development of clinical training for medical school graduates; setting standards for the content of professional and specialized medical training; and establishing the foundations for evaluating and re-evaluating doctors at different levels: graduates, postgraduates and lifelong learners.

Recognizing the need to maintain the professional skills of its medical workforce, the Egyptian government introduced legislation in 2018 to establish mandatory minimum thresholds of engagement in continuing professional development as a prerequisite for the renewal of medical licenses.^9^ The minimum set threshold was suggested to be around 200 to 250 credits on a 5-year cycle (1 credit = 1 hour of educational activity). This target aligns with a variety of international regulatory systems, including that of the United States of America.^10^^,^^11^

A new law was passed in 2019 requiring mandatory renewal of medical practice licences every 5 years.^9^ This mandate was to be applied to recent medical graduates who passed the Egyptian medical licence examination that was first offered in February 2021. Collection of continuing professional development credits will be obligatory for physicians. Egyptian physicians working abroad must collect continuing professional development points for renewal of their licence to practice there. These physicians can attend accredited continuing professional development activities in Egypt. The aim of the law was to motivate physicians to attend accredited continuing professional development activities.

## Framework 

In February 2018, to fulfil the recommendations of the expert group, the continuing professional development committee was established as an independent body working under the regulatory authority. The committee is responsible for setting the accreditation standards; monitoring continuing professional development activities; auditing and reporting the implementation of these activities to the authority’s governing board; updating its role in response to the needs of the public and professionals; and benchmarking its activities against global standards. The committee comprises 11 medical education experts drawn from universities, the health ministry and the Egyptian army. The committee started by exploring the different types of accreditation system present in other countries, comparing provider-based and activity-based systems. Because of the expected large number and types of applicants from the health-care professions, the decision was to start as a provider-based accreditation system for health-care professionals, beginning with physicians, that will facilitate handling accredited providers and monitoring their products. This approach is similar to the continuing professional development systems implemented in Europe,^12^ Oman,^13^ West Bank and Gaza Strip,^14^ USA^10^ and Sudan.^15^ The committee started to accredit online learning activities submitted by either individuals or accredited continuing professional development providers, provided they use an electronic learning (e-learning) platform accredited by the regulatory authority. In general, the continuing professional development system in Egypt follows most of the guidelines of the European Accreditation Council for Continuing Medical Education as a model reference.^12^

## Provider standards

The first continuing professional development by-laws in Egypt were published in August 2018.^16^ A set of eligibility standards, an application process and review process were established under the regulatory authority.

In July 2019, an education and training foundation, the Egyptian Neonatal Network, became the first accredited continuing professional development provider. Only accredited providers are permitted to submit their activities for accreditation. The eligible providers of continuing professional development can be higher education institutions, medical associations, health professional education and publishing companies, governmental agencies, health-care facilities, medical foundations that conduct education and training, and not-for-profit and nongovernmental organizations (NGOs). Any entity with commercial interests, such as pharmaceutical companies and conference organizing agencies, cannot be accredited as a continuing professional development provider.

In June 2020, the by-laws for accreditation of online learning platforms for continuing professional development were published.^17^ The e-learning platform of the medical syndicate was the first to be accredited.^18^ The syndicate has provided 15 online accredited professional development activities in the field of radiology, infection control, cardiology and soft skills, such as leadership, team management and communication skills, for physicians

To avoid conflicts of interest, the continuing professional development committee does not create its own educational programming and therefore does not accredit its own activities.^16^

**Fig. 1:**
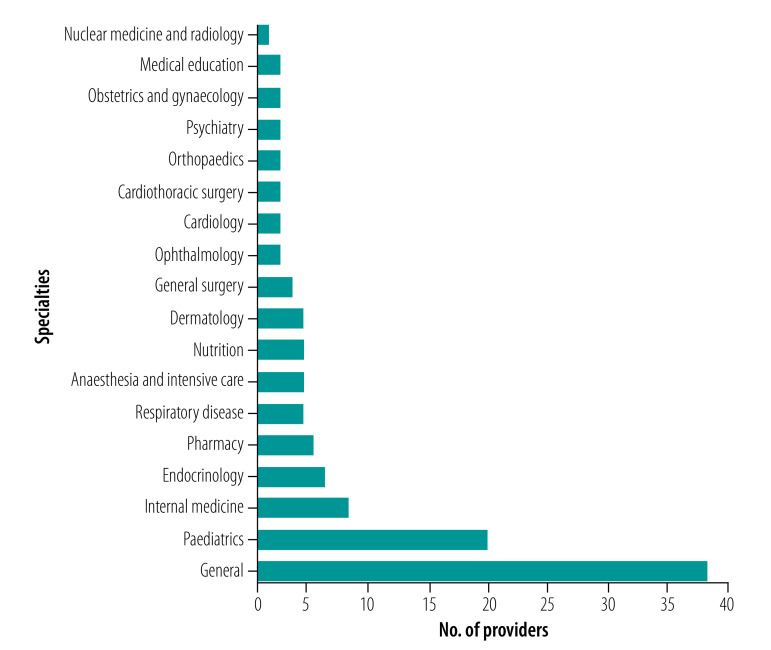
Number of providers of continuing professional development by specialty, Egypt, 2019–2022

Note: Data are for January 2019 to March 2022. General providers include those that offer activities in different specialties, such as schools of medicine.

As of March 2022, 112 organizations have achieved initial accreditation: 72 (64%) medical associations, 20 (18%) higher education institutions, 14 (13%) medical foundations or NGOs, and 6 (5%) health-care facilities. Their distribution by specialty is shown in Fig. 1. Apart from general providers, the providers cover 17 specialties including four major specialties (internal medicine, surgery, obstetrics and gynaecology, and paediatrics) and 13 sub-specialties. The highest number of specialty providers come from paediatrics. General providers include those that offer activities in different specialties, such as schools of medicine. Some specialties (such as clinical pathology, nephrology and pathology) are not covered by dedicated providers although their activities may be offered by general providers.

Most accredited continuing professional development providers are based in the capital city, Cairo. One Egyptian-accredited provider is based in Beirut, Lebanon. Only four providers belong to the pharmacy sector. Two of them are regional organizations: the Arab Board of Pharmacy Specialties and the Union of Arab Pharmacists. The largest national training centre of the Egyptian health ministry – the Princess Fatma Academy for Continuing Professional Education – is also accredited.

Since the launch of the continuing professional development system in August 2018, 154 accredited continuing professional development activities were delivered, with continuing professional development hours ranging from 1 to 30 hours. By March 2022, the continuing professional development hours provided totalled 1076 hours. The precise number of trainees attending all accredited events is not available at this stage. However, we plan to collect more data about participants as part of an assessment of the continuing professional development system. 

By the beginning of 2020, the continuing professional development committee announced the Provider of the Year Award for continuing professional development. The criteria for the award include the number of accredited continuing professional development activities, continuing professional development hours and compliance with the continuing professional development by-laws. Award winners receive a certificate, 50% discount on their fees to the regulatory agency for the next year, and promotion on social media. The Egyptian medical syndicate was rewarded twice, for the years 2020 and 2021.^19^

## Activities standards

### Needs assessment

Continuing professional development activities must be designed to meet the demonstrated educational needs of physicians, with documentation of the needs assessment. Currently, needs assessment in Egypt is completed by the leaders of governing bodies and clinical departments in schools of medicine and hospitals^5^ and scientific committees, which organize the teaching activities based on their perception of the needs of the target audience. We aim for the training needs assessment to be tailored to general physicians and different specialties and directed to the most common diagnoses and prevalent diseases.

### Instructional design

To allow broad and mixed models of educational delivery the professional development standards in Egypt are designed towards learning outcomes rather than an educational format. Learning outcomes are not formally specified but are mentioned by the provider when they submit their activities. We plan to set up intended learning outcomes for continuing professional development in general in collaboration with providers. Currently the primary method of teaching being used by the accredited providers is lectures to large audiences; hands-on workshops are also commonly used. Online teaching has been increasing, especially during the coronavirus disease 2019 pandemic. Accredited providers make considerable effort to develop online education programmes and set up e-educational platforms. The regulatory authority has created inclusive educational design standards to meet the diverse needs of physicians, including those practising in remote areas, and focusing on local health problems and endemic diseases. Delivering professional development activities to those doctors in remote areas could also be achieved by using a mobile simulator training unit, e-learning and online continuing professional development activities.

### Evaluation

For now, the accreditation standards do not oblige providers of continuing professional development to evaluate individual learners or activities. Some providers use pre- and post-tests to measure changes in the knowledge and skills of the participants. We anticipate that when the system is more mature the requirements for accreditation will include evaluation of outcome measures, such as physicians’ self-reflection on their performance, patient care or population health.^20^

### Funding and commercial support

Funding for setting up the continuing professional development system has come from government. We do not have more details available at this stage due to changes to the administrative and financial structure of the regulatory authority that are in progress. Funding of continuing professional development activities can be derived from provider budgets, programme registration fees or appropriate sponsors. The regulatory authority’s by-laws state that pharmaceutical companies, device manufacturers and other commercial organizations cannot use funds to promote their products or services in any continuing professional development content.^16^ Funding from industry is limited to unrestricted educational grants. All funds from a commercial source should be in the form of an educational grant payable to the institution or organization sponsoring the continuing professional development activity, with no stipulations attached, such as selecting faculty, authors, participants, or any matters related to the content. It is acceptable to designate an unrestricted educational grant to a specific event. While there are no detailed regulations on commercial support, the regulatory authority anticipates introducing expectations aligned with the USA’s Accreditation Council for Continuing Medical Education standards for integrity and independence. One challenge to the funding system is that industry bodies pay for the travel and registration of physicians to incentivize and reward their prescribing behaviours. However, in addition to being an unethical practice, the rewards are directed to senior physicians, which deprives others from attending accredited continuing professional development activities.^16^

## Credit standards

The recording of continuing professional development in Egypt uses the credit point-based system. A continuing professional development credit (1 point) equals 1 hour of educational activity. However, a different relationship between time and educational credit may be present in different countries^21^^–^^24^ and this difference should be considered when evaluating credits awarded by international accreditors. The continuing professional development standards classify learning activities into three categories: external, internal and personal credits.^16^ Up to now, any categories of credit can be accumulated; there is no requirement for credits within specific categories. External credit sessions are usually presented by external speakers or guests at a hospital and tend to involve larger groups of practitioners. Internal credit refers to a hospital’s own employees and staff with smaller groups. Personal credit involves self-directed learning activities such as reading, medical writing or completing activities that are not from accredited providers. This credit system matches with the practice in Europe^12^ and USA.^10^ Accredited providers are obliged to retain records of the attendance of its learners for 5 years. Tracking individuals’ continuing professional development performance requires physicians to submit their self-assessment forms and personal development plans to their e-portfolio on the regulatory authority’s website.

In some countries,^13^^–^^15^ the continuing professional development system defines the credit point requirements differently for each health-care specialty (physicians, pharmacists, dentists, nurses and allied health professions) and the distribution of its types within each 3-year continuing professional development cycle. However, the system in Egypt has not yet specified the number of credit points for other health-care specialties. The renewal of licences and the continuing professional development cycle will be every 5 years. 

In Oman^13^ and West Bank and Gaza Strip^14^ these credit points or hours are used as one of the criteria for renewal of medical licenses or contracts; for staff promotion and performance appraisals; for applying for higher studies; or for sponsorship to attend international conferences. In Egypt, the continuing professional development system encourages the same practice and recommends its use in universities.

## Accreditation process

Organizations in Egypt apply for accreditation using the resources of the regulatory authority’s website and an application form. Organizations that can meet the accreditation standards are awarded a 2-year term. Accredited providers are evaluated during their term and at reaccreditation by the regulatory authority. Methods of evaluation include random attendance at accredited professional development events by a representative of the regulatory authority; reviews of submitted post-event documents;^16^ and participants’ feedback on social media and websites. According to the continuing professional development by-laws, the regulatory authority ensures that inquiries into or complaints about continuing professional development providers are handled appropriately to be fair to the provider and the complainant. The regulatory authority can initiate its own inquiries. Any violation of by-laws may lead to a penalty to the provider.^16^ Penalties range from being barred from applying for accreditation in the future, financial penalties or even potentially legal action. 

## Challenges

The system of continuing professional development in Egypt is still in the early stages of development. Despite regulatory and administrative support, the implementation of the system has faced several barriers. While there are several large and sophisticated professional medical societies, opportunities for workplace learning and interprofessional learning are scarce and there is a lack of suitable training venues and equipment for hands-on training. There is no mechanism to fund engagement in continuing professional development without commercial support, and physicians seem reluctant to pay for their own professional development. In focus group discussions during academic and medical society board meetings, senior physicians were especially sceptical of the value of a new continuing professional development system that does not include paid study leave or a travel allowance, and one that restricts funding from the pharmaceutical industry. We plan to assess how different categories of health-care professions perceive the new system.

Allocating time for continuing professional development is the responsibility of the individual physician and is not provided for in standard contracts of work. Many physicians must work extra time to increase their income, which limits their time available for continuing professional development. Every accredited health-care facility must provide a minimum number of internal continuing professional development hours annually as a prerequisite for providing these free of charge to employees, supported by the national health insurance services. We should encourage activities that are conducted inside the workplace, that use different forms of teaching, and that provide allocated, paid time for continuing professional development. Such a development will require improvements in the training infrastructure in health-care facilities and additional support for online education. Mobile training units, delivering workplace training events, should be encouraged to support physicians to attend training.

## Next steps

The migration of health-care professionals across countries for work makes the mutual recognition of continuing professional development credits between countries essential for them.^25^ The regulatory authority of Egypt is currently working on agreements with global organizations, notably the International Academy for Continuing Professional Development Accreditation,^26^ that will facilitate international recognition of the continuing professional development system in Egypt. Leaders of health-care authorities and organizations, medical societies and policy-makers should facilitate effective learning settings to foster a culture of lifelong learning, which lies at the foundation of continuing professional development.^27^

While still in its early stages, the continuing professional development system in Egypt has achieved several milestones, including the establishment of technical and financial by-laws; implementation of a system benchmarked against international standards; recruitment of providers and rejection of unsuitable applicants; the introduction of a system of programme accreditation; and fostering an awareness of a continuing professional development culture in medicine.

Interprofessional continuing professional development, when individuals from many professions learn together, is still rare in Egypt. However, the accreditation of four providers from the pharmacy sector is a positive step towards establishment and extension of continuing professional development to other health professionals. Later in 2022, the Egyptian Health Council^28^ will replace the Compulsory Egyptian Medical Training Authority and will be under the authority of the President of Egypt. All the constituents of the regulatory authority, including its continuing professional development committee, will be transferred to the Egyptian Health Council. This move aims to strengthen, support and stabilize the accreditation system. Because of the large numbers of physicians in the country, the regulatory authority started its work with continuing professional development for physicians. However, the Egyptian Health Council will also control the in-service clinical practice of all health-care professionals. Establishment of an independent body to regulate continuing professional development aims to prevent the issue of different authorities attempting to take over the governance of continuing professional development accreditation in Egypt. The situation differs in some nearby countries and territories, such as Sudan,^15^ and the West Bank and Gaza Strip^14^ where the governing body of continuing professional development accreditation is working under the health ministry, while in Oman^13^ the authority works under the medical and health specialties board, separate from the health ministry.

## Conclusions

The foundations for an effective continuing professional development system have been established in Egypt. Because of increasing cross-country mobility of health-care professionals and patients, the need to ensure high quality and safe patient care has been growing. This development requires political and governmental support, which has now been achieved in Egypt, together with the establishment of an independent accreditor. The new regulatory authority has successfully created standards for continuing professional development, and for an accreditation process and has built up a pool of accredited providers. The public and health-care professionals can anticipate continued evolution in their learning environment to achieve international standards.
